# Public’s perception of policies reducing tobacco availability by regulating the tobacco retail environment: A case study in Egypt

**DOI:** 10.18332/tpc/197384

**Published:** 2025-01-17

**Authors:** Raouf Alebshehy, Eman H. Elsebaie, Oliver Razum

**Affiliations:** 1 School of Public Health, Bielefeld University, Bielefeld, Germany; 2 Department for Health, University of Bath, Bath, United Kingdom; 3 Public Health and Community Medicine Department, Faculty of Medicine, Cairo University, Giza, Egypt

**Keywords:** regulations, tobacco industry, public opinion, retail, tobacco

## Abstract

**INTRODUCTION:**

The tobacco industry presence in the retail environment ensures its access to current and potential tobacco users. Reduction of tobacco retail is an emerging tobacco control measure. Many policies that would potentially lead to reduction in retail are not covered by international tobacco laws and are individually adopted in some jurisdictions. This study examines public perception on potential effect of suggested policies in one of the few countries where tobacco market is increasing, Egypt.

**METHODS:**

A cross-sectional online survey study was implemented in Egypt, June 2023 to March 2024 to assess the perception of the public towards 12 suggested policies that aim to reduce both tobacco availability and purchase by regulating the tobacco retail environment. The survey was disseminated through social media and 320 completed responses were received. Responses on perception followed five-point Likert scales.

**RESULTS:**

Participants felt they could easily access tobacco products around places of residence, work, and study. The largest proportion of participants (53.1%) reported good agreement that the suggested policies would have an impact in reducing tobacco availability and retail, while 39.7% had fair agreement, and only 7.2% had poor agreement.

**CONCLUSIONS:**

Most participants believe that policies reducing tobacco availability and sales would be effective. Non-users rated these policies as more effective than tobacco users. Younger adults viewed these policies more favorably than older adults. The study suggests public support for tobacco control measures to address the prevalent tobacco retail environment in Egypt, indicating a need for policymakers to adopt these measures to protect future generations. Additional research using larger randomly selected populations in Egypt is needed to corroborate these results.

## INTRODUCTION

The tobacco industry understands the marketing importance of making its products available and accessible^1^, spending billions of dollars to ensure its presence in the retail environment^2^. Public health stakeholders recognize that reducing tobacco supply is an evident measure within tobacco control to reduce tobacco use. The World Health Organization Framework Convention on Tobacco Control (WHO FCTC) provides a set of measures for reducing tobacco supply^3^.

The literature shows that measures to reduce tobacco supply extend beyond the treaty requirements, with countries adopting various strategies. A recent scoping review identified sixteen potential policies that restrict tobacco supply by regulating its retail environment. Out of these sixteen policies, twelve have either been individually adopted or studied at country level and are not covered by the international tobacco control treaty. The implementation of these measure varies, with those covered by the treaty being more widely implemented^4^.

Co-creation of public health interventions with involvement of end users, and tailoring interventions to local context is expected to increase the likelihood of developing more effective and sustainable interventions that address lifestyle behavior change^5^. This study, therefore, aims to explore public’s perception on the suggested policies. It examines the accessibility of tobacco products around the work, study, and home settings of the adult population, and assesses public perception of the twelve suggested policies in terms of reducing tobacco availability and purchasing.

Egypt is selected as a setting for the case study considering that it is one of only six countries in the world where tobacco use is still rising, with currently a quarter of its adults using tobacco and half of its population potentially exposed to secondhand smoke. Current data projects an increasing trend, with estimated 27.3% tobacco use by 2030, 54.2% males and 0.3% females^6^. Egypt has been experiencing an expansion of presence of the transnational tobacco industry. In 2012, Japan Tobacco International acquired Egypt’s leading waterpipe company^7^, and by 2024, Philip Morris International expanded its presence in the country by acquiring stocks in Egypt’s Eastern Company, which used to hold a monopoly for decades, and in United, the newly licensed company for tobacco production in Egypt^8^. Egypt has been a Party to the WHO FCTC since 2005 and is therefore required to implement the measures covered by the treaty^9^. Hence, this study focuses on the twelve policies not covered by the treaty. This study further aims to assess the perception of the public towards the suggested policies that aim to reduce both tobacco availability and purchase by regulating the tobacco retail environment.

## METHODS

### Sample

We conducted a cross-sectional online survey study in Egypt. A purposive quota non-probability sampling technique was used to recruit participants. Data were regularly monitored to ensure the sample represents national proportions of tobacco prevalence in Egypt (18.7–30.7% with point estimate 24.7%). Based on evidence from a previous similar study on the estimated perception of the public towards the retail policy interventions^10^, the formula:

$$\text{n}={\text{Z}}_{\alpha }^{2}\times \text{p(}1-\text{p)}/\delta^{2}$$

was used to calculate the sample size (n) of this crosssectional survey, assuming α=0.025, Z_α_=1.960 for 95% confidence level, total width of confidence interval δ=0.1, and an estimated proportion (p) of support equal to 75% of participants. The minimum required sample size for this study was estimated to be 306 participants^11^.

### Ethics

The Ethics Committee of Bielefeld University has reviewed the study protocol and approved the study, as described, as ethically appropriate. Objectives of the study were explained to the participants (by a statement before initiation of the online survey) and they were completely free to accept or refuse participation. Starting the survey was considered consent to participate in the study. Strict confidentiality about participants’ personal data, secured by the questionnaire being anonymous, was maintained throughout data collection, entry, and analysis.

### Tool

An anonymous structured questionnaire was designed on UniPark based on the findings of a scoping review of retail environment policies to reduce tobacco availability. It collected demographic characteristics of participants in addition to data on status of tobacco use and the degree of accessibility to tobacco, the likelihood of suggested policies in reducing tobacco availability, and the likelihood of them in reducing tobacco purchase. Responses on perception followed a five-point Likert scale: 5=strongly agree, 4=agree, 3=neutral, 2=disagree, and 1=strongly disagree. The questionnaire was assessed for content validity with an expert panel of 3 researchers with knowledge and expertise on tobacco/public health research. The experts were asked to individually review the relevance of the items, and the survey was adjusted accordingly as per their feedback^12^. After assessment of content validity, the final version of the questionnaire was translated into Arabic followed by a back-translation into English performed by two additional translators. The back translators compared their translations with the previous English version. Any discrepancies that were identified were resolved by discussions between the researchers and the translators^13^. The survey questionnaire is given in the Supplementary file.

### Data collection

The survey was disseminated electronically through social platforms targeting the Egyptian population. A page named Tobacco and Smoking Control Studies was created with a sponsored advertisement of the study, including the main information, targeting the adult population of Egypt. Participants’ enrolment was monitored weekly to assess the proportions enrolled according to the sampling scheme during the data collection period. A total of 2837 participants opened the survey, with a completion rate of 11.2%, and 320 completed participations were collected.

### Analysis and synthesis

The data were coded and cleaned on a data sheet prepared in Excel. Microsoft Excel 2016 was used for data entry, and the Statistical Package for Social Sciences (SPSS version 24) was used for data analysis. All collected data were revised for competencies and logical consistency. Data exploration, as usual, or skewed distribution, was done using Kolmogorov– Smirnov/Shapiro–Wilk’s test. Simple descriptive statistics of mean and standard deviation was used for the summary of numerical variables, while frequencies and percentages were used for categorical ones. A comparison of proportions was performed using the chi-squared test to present participants’ characteristics distribution with tobacco use status (current users and non-users). One-way ANOVA test was used to compare the mean scores of degrees of accessibility to tobacco and perception of potential policies’ reduction of tobacco availability and purchase with the tobacco use status, sex, and different age categories. A p<0.05 was considered significant.

The results are tabulated into participants’ characteristics in terms of sex, age groups, and professional status and their distribution within each tobacco use status. Accessibility and perception results were tabulated to present the total sample mean scores of degrees of accessibility to tobacco and perception on potential policies’ reduction of tobacco availability and purchase, and mean score comparisons with age, sex, and tobacco use. The total perception score was categorized into three levels: poor, fair, and good agreement. The categorization was based on percentiles, with scores below the 50th percentile considered poor, scores between the 50th and 75th percentile considered fair, and scores equal to or above the 75th percentile considered good.

## RESULTS

A total of 320 participants took part in this study, with characteristics summarized in Supplementary file Table 1. The prevalence of tobacco use among the participants was 22.8%, which reflects the actual prevalence of tobacco use in Egypt.

Accessibility and perception results are presented in Table 1. The survey data suggest that overall, participants agreed with the statements that they could easily get tobacco products around their work/study place and home, indicating high perceived availability of tobacco, especially around residential areas. Regarding the public perception of the likelihood that the suggested policies would reduce tobacco availability and purchase, the largest portion (53.1%) had a good agreement that the policies would have an impact if enacted. Meanwhile, 39.7% had a fair agreement, and only 7.2% had a poor agreement.

**Table 1:** The mean scores of degrees of accessibility to tobacco and perception on potential policies’ reduction of tobacco availability and purchase

		*Tobacco use status*			*Sex*			*Age (years)*							*Total*
		*users*	*Nonusers*	*p*	*Male*	*Female*	*p*	*18–24*	*25–34*	*35–44*	*45–54*	*55–64*	*≥65*	*p*	
**Accessibility to tobacco products**															
I can easily get tobacco products around my work/study place if I want to		3.79±1.24	3.76±1.40	0.864	3.87±1.33	3.59±1.41	0.075	3.66±1.47	3.75±1.24	3.86±1.36	4.06±1.01	3.80±1.13	3.0±2.82	0.646	3.77±1.37
I can easily get tobacco products around my home if I want to		4.12±1.02	4.06±1.31	0.711	4.21±1.19	3.86±1.32	0.017*	4.08±1.38	4.04±1.16	4.02±1.26	4.33±0.66	3.80±1.13	5.0±0	0.698	4.08±1.25
**Perception on potential policies’ reduction of tobacco availability and purchase**															
Restricting tobacco retail outlets per density of population	Availability	3.32±1.34	3.76±1.12	0.013*	3.57±1.26	3.81±1.03	0.055	3.70±1.13	3.78±1.11	3.68±1.21	3.33±1.30	3.6±1.43	3±2.83	0.613	3.67±1.18
	Purchase	3.13±1.44	3.32±1.18	0.302	3.28±1.31	3.27±1.12	0.947	3.26±1.18	3.31±1.21	3.45±1.26	2.8±1.27	3.4±1.51	3±2.83	0.253	3.28±1.24
Banning home delivery of tobacco	Availability	3.53±1.34	3.71±1.28	0.292	3.58±1.37	3.82±1.16	0.086	3.47±1.37	3.73±1.18	4.04±1.14	3.3±1.32	3.8±1.4	4±1.41	0.017*	3.68±1.30
	Purchase	3.09±1.43	3.51±1.27	0.028*	3.33±1.40	3.54±1.17	0.164	3.28±1.34	3.34±1.20	3.8±1.25	2.86±1.31	3.7±1.25	3±2.83	0.008*	3.42±1.32
Banning tray/mobile tobacco sale	Availability	3.45±1.34	3.91±1.16	0.008*	3.68±1.31	4.02±1.03	0.010*	3.80±1.23	3.87±1.17	3.92±1.19	3.43±1.28	3.7±1.49	4±1.41	0.554	3.81±1.22
	Purchase	3.09±1.42	3.64±1.22	0.003*	3.41±1.37	3.68±1.14	0.058	3.5±1.34	3.53±1.21	3.64±1.25	3.13±1.22	3.7±1.49	4±1.41	0.543	3.52±1.29
Capping the tobacco amount allowed per purchase	Availability	3.78±1.29	4±1.11	0.155	3.90±1.23	4.01±1.03	0.402	3.92±1.17	4±1.0	4.07±1.18	3.73±1.05	4±1.41	2±1.41	0.156	3.95±1.16
	Purchase	3.72±1.37	3.95±1.11	0.185	3.84±1.29	4.00±0.97	0.194	3.88±1.22	3.73±1.18	4.05±1.14	3.83±0.99	4.1±1.20	2±1.41	0.153	3.91±1.18
Requiring a minimum distance between tobacco retailers	Availability	3.45±1.38	3.74±1.18	1	3.62±1.32	3.77±1.09	0.290	3.53±1.22	3.78±1.27	3.91±1.19	3.46±1.22	3.9±1.37	3±2.83	0.186	3.68±1.24
	Purchase	3.02±1.39	3.53±1.23	0.003*	3.36±1.37	3.5±1.12	0.332	3.30±1.27	3.21±1.24	3.68±1.27	3.13±1.25	4.1±1.29	3±2.83	0.058	3.42±1.28
Banning tobacco retail outlets in or within a minimum distance from specific facilities	Availability	3.67±1.28	4.21±1.0	0.001*	4.05±1.14	4.15±1.0	0.402	4.11±1.07	4.24±0.99	4.07±1.13	3.9±1.12	3.9±1.37	4±1.41	0.833	4.09±1.09
	Purchase	3.41±1.35	3.97±1.09	0.001*	3.86±1.21	3.81±1.13	0.718	3.92±1.16	3.70±1.15	3.85±1.23	3.56±1.22	4.2±0.92	4±1.41	0.583	3.85±1.18
Banning tobacco sale in specific retail outlets	Availability	3.84±1.23	4.34±0.92	0.002*	4.21±1.11	4.26±0.86	0.685	4.44±0.89	4.12±1.0	4.11±1.09	3.96±1.16	3.7±1.34	4±1.41	0.026*	4.23±1.02
	Purchase	3.69±1.29	4.18±0.96	0.004*	4.05±1.15	4.10±0.91	0.629	4.28±0.97	3.82±1.07	4±1.11	3.66±1.18	3.9±0.88	4±1.41	0.021*	4.07±1.06
Restricting tobacco retail outlets per geographical area	Availability	3.60±1.39	4.15±0.93	0.002*	3.96±1.19	4.13±0.087	0.132	4.15±0.98	4.07±1.08	3.98±1.13	3.7±1.12	3.7±1.34	3±2.83	0.175	4.03±1.08
	Purchase	3.45±1.46	3.96±1.01	0.006*	3.82±1.26	3.88±0.93	0.614	3.96±1.09	3.65±1.15	3.86±1.18	3.53±1.14	3.9±1.20	3±2.83	0.306	3.85±1.14
Government controlled outlets	Availability	3.53±1.46	3.82±1.21	0.129	3.72±1.37	3.81±1.12	0.526	3.88±1.20	3.70±1.25	3.67±1.35	3.73±1.36	3.2±1.40	2.5±2.12	0.329	3.76±1.28
	Purchase	3.38±1.48	3.64±1.23	0.167	3.59±1.37	3.57±1.16	0.877	3.66±1.28	3.53±1.19	3.56±1.34	3.46±1.38	3.5±1.18	2.5±2.12	0.811	3.59±1.29
Limit the number of hours or days in which tobacco can be sold	Availability	3.49±1.41	3.83±1.21	0.066	3.73±1.32	3.78±1.17	0.699	3.78±1.21	3.85±1.26	3.78±1.27	3.56±1.38	3.3±1.49	2.5±2.12	0.500	3.75±1.26
	Purchase	3.30±1.48	3.70±1.25	0.039*	3.60±1.41	3.62±1.16	0.880	3.69±1.28	3.53±1.29	3.63±1.35	3.4±1.43	3.4±1.35	2.5±2.12	0.684	3.61±1.32
Reducing tobacco products availability and proximity within a retail outlet	Availability	3.61±1.37	4±1.02	0.026*	3.85±1.21	4±0.94	0.243	3.98±1.02	3.85±1.06	3.91±1.21	3.76±1.19	3.8±1.40	2.5±2.12	0.476	3.91±1.12
	Purchase	3.54±1.40	3.92±1.02	0.036*	3.82±1.22	3.85±0.97	0.845	3.95±1.07	3.53±1.12	3.85±1.18	3.73±1.11	3.9±1.20	2.5±2.12	0.188	3.84±1.13
Banning tobacco sale of one or more tobacco products	Availability	3.76±1.22	4.21±1.0	0.002*	4.10±1.14	4.11±0.95	0.941	4.21±1.0	4.07±1.10	4±1.10	4.13±1.01	3.9±1.29	3±2.83	0.407	4.11±1.07
	Purchase	3.60±1.30	4.14±1.01	0.001*	4.02±1.19	4.02±0.96	0.996	4.21±1.02	3.73±1.29	3.92±1.08	3.86±1.14	4.1±0.99	3±2.83	0.064	4.02±1.11
**Total perception score**		83.56±26.10	93.26±18.88	0.004*	90.07±23.10	92.63±17.33	0.260	91.97±19.02	89.78±19.01	92.53±23.75	85.03±21.59	90.4±24.35	74±50.91	0.464	91±21

On a scale from 1 = ‘strongly disagree’ to 5 = ‘strongly agree’, with a midpoint of 3 = ‘neutral’.

* Significant p<0.05.

** One-way ANOVA test.

Regarding the perceived effectiveness of specific tobacco control policies in reducing the availability of tobacco, highly perceived policies included banning tobacco sale in specific retail outlets, banning tobacco sale of one or more tobacco products, banning tobacco retail outlets in or within a minimum distance from specific facilities, and restricting tobacco retail outlets per geographical area. For the perceived effectiveness of specific tobacco control policies on purchasing tobacco, the highly perceived policies included banning tobacco sales in specific retail outlets, banning tobacco sales of one or more tobacco products, and capping the tobacco amount allowed per purchase.

Non-users had a more favorable view of the potential effects of policies compared to tobacco users. The two sex groups held comparable perspectives on the suite of tobacco control policies presented. The younger adults, particularly those aged <45 years, tended to perceive many policies as effective at limiting tobacco availability and purchase, compared to older adults.

## DISCUSSION

Our study shows that, within the study population, tobacco products are perceived as being easily accessible and available around places of residence, study, and work. This is alarming considering the high percentage of young people contributing to this study and resonates with the documented efforts of the tobacco industry in making its products available and promoted to future generations^1^. The literature suggests that a higher density of retailers reduces the cost needed to get the product and therefore increases purchase opportunities, and while there is more research needed to study the association between tobacco retailer availability and tobacco use, literature suggests a positive association between higher density of retailers around homes and current smoking in adults and adolescents^14^.

Overall, most participants agreed that suggested policies would positively decrease both the availability and purchase of tobacco products if enacted. These echo studies implemented in other countries about the public support for tobacco retail reduction measures^10^. The policy to have the highest perceived potential impact on reducing tobacco product availability and purchase was banning tobacco sales in specific retail outlets. This might reflect the reality of easy access to tobacco products in Egypt through supermarkets, gas stations, kiosks, mobile retailers, tobacco sellers etc. Restricting tobacco retail outlets per density of population, although perceived as effective in reducing tobacco availability and purchase, more by non-users than users, was the lowest mean score among the twelve suggested policies, which might be a reflection of the high density of Egypt’s population, as 95% of its population are reported to live on 5% of its land^15^.

Although non-users of tobacco had a significantly higher score of perception of the suggested policies’ effectiveness compared to tobacco users, both perceived policies as potentially effective. This result and the literature suggestions that retail reduction might decrease the impulse of tobacco purchase, signal to policymakers a positive public perception across the board to the implementation of retail reduction policies^4^. Moreover, our study suggests, within the study population, a comparable perspective among males and females on the suite of tobacco control policies presented.

The total perception score of this study shows that, within the study population, younger adults tend to view many policies as more effective at limiting tobacco availability and purchase than older adults. This positive perception from young adults is a significant finding since most smokers start before the age of 18 years^16^, tobacco use prevalence in Egypt is increasing^6^, and Egypt is one of the few places where the tobacco market is increasing^8^. More value should be given to this finding, recognizing that the tobacco industry is investing in getting young people addicted to secure its future profits^1^.

The strong support within the study population to the potential effects of suggested measures reflects the public’s real need and desire to address the normalized tobacco retail environment. A quarter of the Egyptian population is using tobacco, half of the public is exposed to secondhand smoke, and it is almost impossible for children not to be exposed to tobacco products or their advertisements. While the tobacco industry sees the future Egyptians as a potential source of profit, policymakers should respond to the publicly perceived effective tobacco control measures and step up their efforts to protect future generations by adopting population-supported measures.

### Limitations

This study shows the potential public support for tobacco reduction measures by regulating the retail environment. However, due to its cross-sectional design, it cannot attribute causality. The convenience sample through an online survey is subject to response bias. The convenience sample does not allow for the results to be attributable to the general population of Egypt, which would need a randomly selected stratified sample.

## CONCLUSIONS

Previous work scoped potential policies for reducing tobacco by regulating the retail environment^4^. This study builds on that by assessing the perception of the Egyptian public on the potential effects of identified policies on the availability and purchase of tobacco. The findings are consistent with studies elsewhere in presenting strong support for the potential effectiveness of tobacco retail environment regulations, with the public perception that such policies would impact both the availability and purchase of tobacco in Egypt. Considering that these policies are not fully covered by either the WHO FCTC or the current Egyptian tobacco policies, decision-makers within Egypt and international regulators should note and consider these policies and their potential impact. Egypt is encouraged to go ahead with the policies that gained strong support from the public when supported by evidence on effectiveness.

## Supplementary Material



## Data Availability

The data supporting this research are available from the authors on reasonable request.
