# Supplementary material for: Public’s perception of policies reducing tobacco availability by regulating the tobacco retail environment: A case study in Egypt
Source: Tob Prev Cessat. 2025 Jan 17;11:10.18332/tpc/197384. doi: 10.18332/tpc/197384 (PMC11740601; doi:10.18332/tpc/197384)
Supplement: Supplementary file 1 [file TPC-11-06-s1.pdf]

**Supplementary file**

**Supplementary table 1: Characteristics of the study participants**

| | **% of total participants** | **Tobacco users (22.8%)** | **Non-users (77.2%)** |
|---|---|---|---|
| **Sex** | | | |
| • Male | 61.9% | 79.5% | 56.7% |
| • Female | 38.1% | 20.5% | 43.3% |
| **Age group** | | | |
| • 18-24 | 44.4% | 17.8% | 52.2% |
| • 25-34 | 12.8% | 15.1% | 12.1% |
| • 35-44 | 29.7% | 49.3% | 23.9% |
| • 45-54 | 9.4% | 13.7% | 8.1% |
| • 55-64 | 3.1% | 2.7% | 3.2% |
| • 65 and over | 0.6% | 1.4% | 0.4% |
| **Profession status** | | | |
| • Student | 43.1% | 15.1% | 51.4% |
| • Working | 49.4% | 78.1% | 40.9% |
| • Unemployed | 7.5% | 6.8% | 7.7% |

**Questionnaire**

**Pre survey text:**

Consent to participate.

By responding to this survey, I agree to take part in a research study that aims to explore public perception on some policies aiming to reduce tobacco availability and tobacco purchasing by regulating tobacco retail environment.

I have been provided with information explaining what participation in this study involves. I understand that taking part in the study is voluntary, the information I provide is anonymous, and that I'm free to withdraw my consent to participate in the study at any time by closing the browser.

I understand that I am free discuss this project with Dr Raouf Alebshehy of Bielefeld University, who I can contact at raouf.alebshehy@uni-bielefeld.de

Joining the survey is considered as a consent to participate in this study. If you are happy to participate, go to the next page.

| | | |
|---|---|---|
| | Recruitment | |
| 1 | How did you find this questionnaire? | 1. Facebook<br>2. Twitter<br>3. Suggested by a friend<br>4. Other (specify) |
| | Participants' Characteristics | |
| 2 | Sex | 1. Male<br>2. Female |
| 3 | Age group | 1. 18-24<br>2. 25-34<br>3. 35-44<br>4. 45-54<br>5. 55-64<br>6. 65 and over |
| 4 | Profession status | 1. Student<br>2. Working<br>3. Unemployed |

©2025 Alebshehy R. et al.
